# A Linear Model for Transcription Factor Binding Affinity Prediction in Protein Binding Microarrays

**DOI:** 10.1371/journal.pone.0020059

**Published:** 2011-05-26

**Authors:** Matti Annala, Kirsti Laurila, Harri Lähdesmäki, Matti Nykter

**Affiliations:** 1 Department of Signal Processing, Tampere University of Technology, Tampere, Finland; 2 Department of Information and Computer Science, Aalto University School of Science and Technology, Helsinki, Finland; 3 Turku Centre for Biotechnology, Turku University, Turku, Finland; Center for Genomic Regulation, Spain

## Abstract

Protein binding microarrays (PBM) are a high throughput technology used to characterize protein-DNA binding. The arrays measure a protein's affinity toward thousands of double-stranded DNA sequences at once, producing a comprehensive binding specificity catalog. We present a linear model for predicting the binding affinity of a protein toward DNA sequences based on PBM data. Our model represents the measured intensity of an individual probe as a sum of the binding affinity contributions of the probe's subsequences. These subsequences characterize a DNA binding motif and can be used to predict the intensity of protein binding against arbitrary DNA sequences. Our method was the best performer in the Dialogue for Reverse Engineering Assessments and Methods 5 (DREAM5) transcription factor/DNA motif recognition challenge. For the DREAM5 bonus challenge, we also developed an approach for the identification of transcription factors based on their PBM binding profiles. Our approach for TF identification achieved the best performance in the bonus challenge.

## Introduction

DNA binding proteins form a diverse class of proteins that play crucial roles in many cellular processes. They replicate and repair the genome, transcribe genes, form the structure of chromatin, and mediate intracellular signals, among other activities [1]. Most such DNA binding proteins have a high degree of specificity toward a particular DNA sequence motif and can interact with other nearby proteins, forming regulatory complexes that control many cellular processes. Transcription factors (TF) are a well known subclass of these proteins: they regulate gene transcription within the nucleus by binding to regulatory sites near gene promoter regions or enhancers [1], [2]. In higher organisms, the promoter region of a gene can contain dozens of bound transcription factors that together control the gene's expression through their interplay. Since TFs are central players in many cellular signaling pathways, they are also associated with a wide variety of diseases, forming an important drug target [3]–[5]. To discover and understand these pathways, it is important to know the target genes of individual transcription factors.

Methods for TF target identification can be divided into two broad classes: methods that observe binding sites directly, and methods that use TF binding specificity models to computationally identify putative binding sites. A traditional method for directly discovering the DNA binding sites of proteins has been to use protein-DNA crosslinking, followed by DNA fragmentation and chromatin immunoprecipitation (ChIP) [6], [7]. In this approach, the bound DNA fragments are analyzed using a technique such as Sanger sequencing, tiled DNA microarrays (ChIP-chip) [8], or high throughput sequencing (ChIP-seq) [9]. The outcome is a map of *in vivo* protein-DNA binding sites in the studied cell population. Since ChIP-based methods characterize protein DNA binding sites *in vivo*, they require that the protein be expressed, nuclear and attached to the chromatin when the cells are fixed. ChIP-discovered binding sites are also cell type specific, due to epigenetic effects. DNA immunoprecipitation (DIP) provides an alternative approach where protein-DNA complexes are immunoprecipitated from a mixture of purified protein and naked genomic DNA. This allows the protein's binding affinity to be measured *in vitro*, without the confounding effect of epigenetics or cellular dynamics. Another benefit of this method is that no crosslinking agent is required [10].

**Figure 1:**
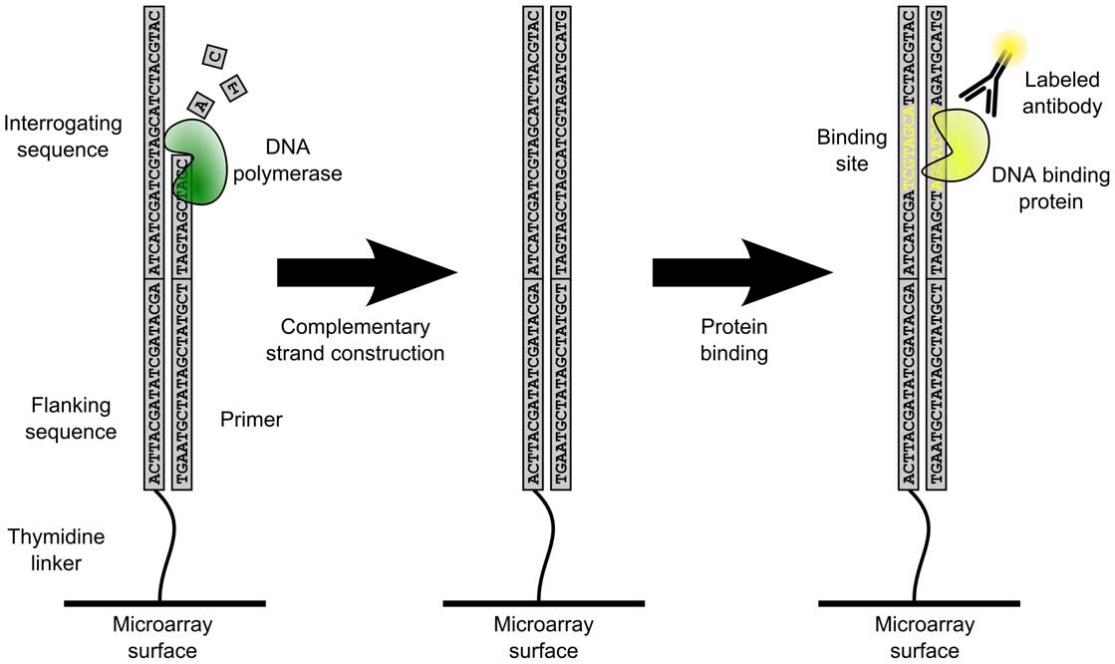
Overview of a PBM array experiment. PBM arrays are constructed by taking a normal oligonucleotide microarray and constructing a complementary strand for each probe using DNA polymerase. Probe-invariant flanking sequences are used as complementary targets for the polymerase primer. Antibody-labeled DNA binding protein is then allowed to bind to probes on the microarray slide, according to the protein's sequence binding preferences.

Protein binding microarrays (PBM) [11], [12] provide a second alternative to ChIP-based techniques. These arrays contain thousands of double-stranded DNA (dsDNA) probes, and analyze a protein's binding specificity toward a large number of DNA sequences at once. Usually the probes are designed so that every K-mer up to a certain length is represented in the sequence of at least one probe on the array [12]. The number of required probes in such designs is often minimized using a graph theoretic method based on de Bruijn sequences [13], [14]. PBM arrays are typically based on a custom single stranded DNA (ssDNA) microarray platform whose probe sequences consist of an interrogating sequence and a flanking sequence. The probe-specific interrogating sequences are designed to cover a maximal number of short DNA sequences, while the probe-invariant flanking sequences are used for complementary primer hybridization. Once the complementary primers have been hybridized onto the flanking sequences, a DNA polymerase is used to extend the primers, producing dsDNA probes that emulate open chromatin [12]. In the measurement phase, the antibody-labeled DNA binding protein is hybridized onto the microarray slide, and will preferentially bind with dsDNA probes containing sequences close to the protein's true binding motif (Figure 1). The microarray slide is then imaged, producing the final spot intensity image. Since PBM arrays interrogate the binding specificity *in vitro*, they are agnostic to epigenetic effects or cellular state.

Binding motifs can be identified from ChIP, DIP or PBM experiments by computationally analyzing the sequence or microarray data produced by the measurement platform. Any discovered motifs are then represented in the form of a motif model for subsequent use in binding site prediction. Commonly used models include consensus sequences, position frequency matrices (PFM) and position weight matrices (PWM) [15], [16]. Of these three, consensus sequences are the simplest, consisting of a short nucleotide string that specifies the nucleotides allowed at each position within the motif. The PFM model builds on consensus sequences by incorporating quantitative information about the relative frequencies of nucleotides at different positions within the motif. The PWM model further generalizes the PFM by replacing the nucleotide frequencies with arbitrary affinity scores, with log-odds often used for the scores [16].

Representation of binding motifs as PFMs or PWMs makes the implicit assumption that all mononucleotides contribute independently to the binding affinity. Studies done on zinc finger proteins have challenged this assumption [17], although other authors have found PWMs to provide a reasonably good approximation of reality [18]. Still, the PWM model currently remains the most commonly used motif model, although the introduction of comprehensive binding site interrogation methods has led some to question whether models more general than PWMs might yield better accuracy in binding site prediction. To this end, some authors have proposed using longer nucleotide subsequences (K-mers) rather than mononucleotide based models [17], [19]. This approach has the benefit of capturing short range interdependencies between nucleotides, but significantly increases the number of variables if both positional information and K-mer sequences are simultaneously included in the model.

The literature describes a number of different algorithms for inferring motif models from binding-enriched unaligned sequences. Lawrence et al. formulate the problem using a model-based approach and develop a Gibbs sampling technique for statistical inference [20]. The MEME algorithm uses expectation maximization (EM) to simultaneously align sequences and discover contiguous PWM motifs of fixed length [21], while Van Helden et al. build consensus sequences based on significantly enriched 6-mers [22]. More recent algorithms place increased emphasis on utilizing quantitative binding affinity measurements: The MDScan algorithm takes a list of DNA sequences ranked according to their expected motif enrichment, and generates a list of seed K-mers based on the sequences. PFMs are then constructed and iteratively updated based on a maximum a posteriori (MAP) scoring function [23]. The MatrixREDUCE algorithm uses a model based on statistical mechanics to fit a PWM to high throughput binding affinity data [24]. Berger et al. use a normalized Wilcoxon-Mann-Whitney statistic to calculate an enrichment score for all 8-mers based on PBM data, and pick the highest scoring 8-mer as a seed sequence. They then determine a final PWM by tweaking the seed sequence and repeatedly calculating the statistic for each variant, giving the algorithm its name “Seed and Wobble” [13]. The RankMotif++ algorithm fits a PWM motif model using a likelihood maximization approach based on relative probe binding intensities [25].

Motif models have been successfully applied in several biological contexts in the past. For example, Litvak et al. recently used PWM motif scanning to predict a feed-forward motif (consisting of NFkB, ATF3 and CEBPd) that was shown to shape the transcriptional response of TLR stimulated macrophages [26], while Segal et al. used a dinucleotide motif model to study nucleosome positioning along DNA [27]. Still, the accurate and objective evaluation of the performance of different binding models remains an open problem. Recently, the DREAM initiative was begun with the aim of establishing a platform for the fair comparison of the strengths and weaknesses of different computational methods. TF binding prediction in particular has been addressed by DREAM on multiple occasions [28], [29].

While the PWM motif model has proven its usefulness in many applications, more general approaches can also be considered. One alternative is the full 8-mer model described by Chen et al. in their RankMotif++ paper. Chen et al. compare the performance of RankMotif++ against a full 8-mer model where the signal intensity of a PBM probe is predicted by taking the 8-mer subsequence with the highest median intensity across the probes containing it on the training PBM array, and using that median intensity as the prediction for the target array [25]. Another recent departure from PWM models is by Agius et al., who use a string kernel and support vector regression to learn a motif model that is then used in binding affinity prediction [30].

We present a new linear motif model that represents a TF's binding affinity toward a DNA sequence as a linear combination of its affinities towards the variable-length K-mers that make up the DNA sequence. Here by “binding affinity” we refer to a quantity that measures the relative specificity of a protein towards a particular DNA sequence. It should be noted that these binding affinities do not directly correspond to dissociation constants or other physical quantities. Our motif model can be learned from any binding affinity data where a binding affinity score is associated with each interrogated DNA sequence. The model produces prediction results better than those produced using full 8-mer models, while having a more compact motif representation. We illustrate the power of our model by applying it to PBM data from the DREAM5 transcription factor/DNA motif recognition challenge [29].

Since we use K-mers rather than mononucleotides, our model can capture full binding specificity information for short motifs (shorter than 9 bases). For longer motifs, our model assumes that binding affinity can be modeled as an additive effect of the component K-mers. Since the additivity assumption has been found to be a good approximation at the mononucleotide level [18], we suspect that the assumption holds even better at the level of K-mers. Our model's quantitative and accurate binding affinity predictions also enable its use in modeling low-affinity interactions, which have been shown to play a significant role in model organisms [31].

In addition to the prediction of binding affinity, an important related problem is the identification of unknown bound TFs. Such a problem can arise, for example, when a set of genes having similar gene expression profiles share a common regulator or when indirect binding sites are found in ChIP experiments. The increasing interest in differential transcriptional regulation between individuals also highlights the importance of TF identification [32]. The identification problem can be approached by using motif discovery tools to find common DNA sequences in genes' promoters and by comparing these motifs to known TF binding target sequences. We demonstrate this approach and show the possibilities and challenges of TF recognition using data from the DREAM5 transcription factor/DNA motif recognition challenge.

## Methods

### DNA binding model

Our binding model represents the measured binding affinity of a protein towards a DNA sequence as the sum of the binding affinity contributions of the sequence's constituent subsequences. The subsequences are allowed to vary in length, so that the affinity contributions of all constituent 4–8-mers are included in the model. Without loss of generality, we can restrict our discussion to the case where the motif model is learned from PBM data, so that the differentially bound sequences come from dsDNA probes.

**Figure 2:**
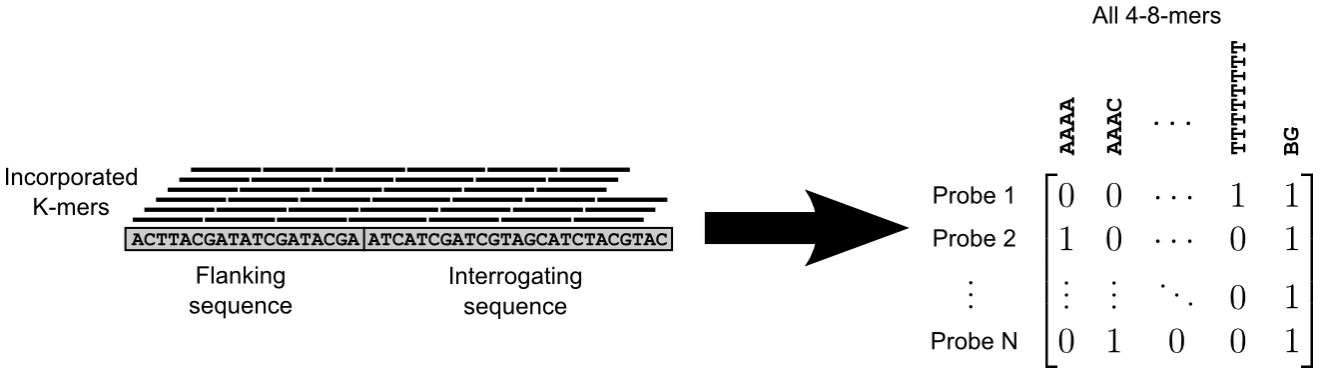
Construction of a PBM array design matrix. Both the flanking and interrogating sequences are considered when building the matrix. A column for a constant background component is also included in the matrix.

As the first step of our algorithm, the K-mers present in the probe sequences on a PBM array are represented as a design matrix ***H***, so that
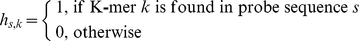
The design matrix is built in a strand specific manner, so that reverse complement K-mers are considered separately. This allows our model to capture strand specific effects. An extra column of ones is also added to the design matrix in order to account for a constant background in the probe intensities (Figure 2).

If a probe sequence *s* contains multiple copies of a K-mer *k*, we set 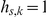. We empirically evaluated the effect of setting 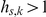 in such cases, but found no benefit. Restricting the design matrix elements to binary values also allows the matrix to be stored in a more compact format.

Once a design matrix has been constructed, we solve the K-mer affinity contributions from the linear system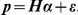.In this model, ***p*** is a vector containing log-transformed and mean-subtracted probe intensities from a PBM experiment, **α** is a vector of K-mer affinity contributions, and ***H*** is the design matrix of the PBM array used in the experiment. The error term ***ε*** accounts for noise in the measured probe intensities. Mean-subtracted probe intensities are used in order to prevent situations where K-mers present in the flanking sequence end up with anomalously high affinities due to them inadvertently modeling the constant background intensity.

### Regularization

If all 4–8-mers are included in the model, the system is underdetermined, having roughly 90 000 unknowns. For this reason, we regularize the system by only including 7–8-mers with the highest median intensity across the probes that incorporated them. We also include all 4–6-mers, since they are critical for accurately predicting the intensities of low affinity probes. This regularization approach is based on the assumption that K-mers with the highest median intensity are the most informative in terms of protein binding.

We also considered regularizing the system by minimizing an 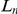-norm of the affinity vector **α**. Ridge regression is one method where the 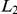-norm is minimized in parallel with the residual. While ridge regression can be implemented efficiently, the 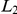-norm is a bad choice for our problem, since one expects **α** to be sparse, a constraint that the 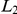-norm does not seek to enforce. Instead, minimization of the 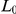
- or 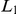-norm is more suitable. However, LASSO [33] and other tested regularization methods did not run in a practical amount of time for a system of this scale. We therefore opted to use the more domain specific regularization technique described above.

### Binding prediction

The sparse but large linear system is solved for the affinity vector by applying the conjugate gradient method to the normal equations
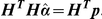
Elements of the affinity vector **α** are not constrained to be non-negative. Adding this constraint would make the system more computationally intensive to solve, and we have no biological reason to assume that specific K-mers cannot actually inhibit the binding of certain proteins.

Once the affinity vector 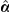 of a protein has been estimated from the data, we can use it to predict binding affinities against arbitrary DNA sequences by constructing another design matrix ***H′*** for the given sequences, and calculating the predicted intensities ***p′*** as
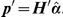
The previously subtracted mean is then added back to the predicted probe intensities. The sequences used in predicting binding intensities should be of the same length as the probe sequences on the PBM array.

### Preprocessing

**Figure 3:**
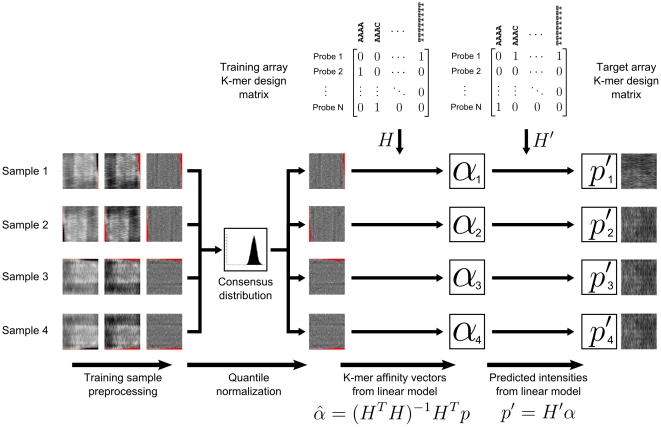
Overview of the full binding intensity prediction model. PBM samples are first preprocessed by removing dark outlier probes and performing spatial detrending. The samples are then quantile normalized before application of the linear model. In this example, the predicted binding intensities are shown to be calculated against probe sequences on another PBM array, but could just as well be calculated for genomic sequences or any other DNA sequences.

If we are dealing with raw PBM array data, we have to preprocess and normalize the probe intensity profiles before solving the affinity vector **α** from the linear system. The full algorithm for learning the affinity vector **α** and predicting binding intensities based on PBM data is shown in Figure 3. The figure includes all preprocessing steps that are applied before the linear model. All steps in the algorithm are applied to log-transformed probe intensities.

**Figure 4:**
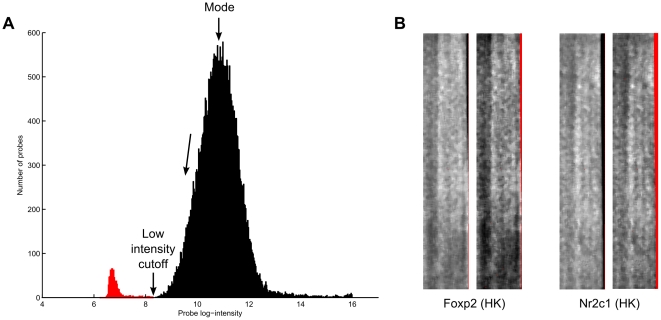
Low intensity probe filtering. (a) A filter cutoff point is determined based on the intensity histogram. (b) Two examples of how low intensity filtering successfully removes dark edge artifacts in PBM samples. In both samples pairs, the original sample is on the left, and the filtered sample on the right. Red pixels indicate missing or discarded intensity values.

In the first preprocessing step of Figure 3, we construct a spatial probe intensity map and intensity histogram for each PBM sample. Probes with very low intensities are then discarded using a threshold derived from the intensity histogram. We calculate the threshold by taking the mode 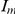 of the histogram, and then move toward lower intensity bins 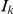 until 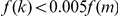, where 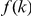 is the frequency in bin *k*, and 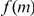 is the frequency at the mode (Figure 4).

**Figure 5:**
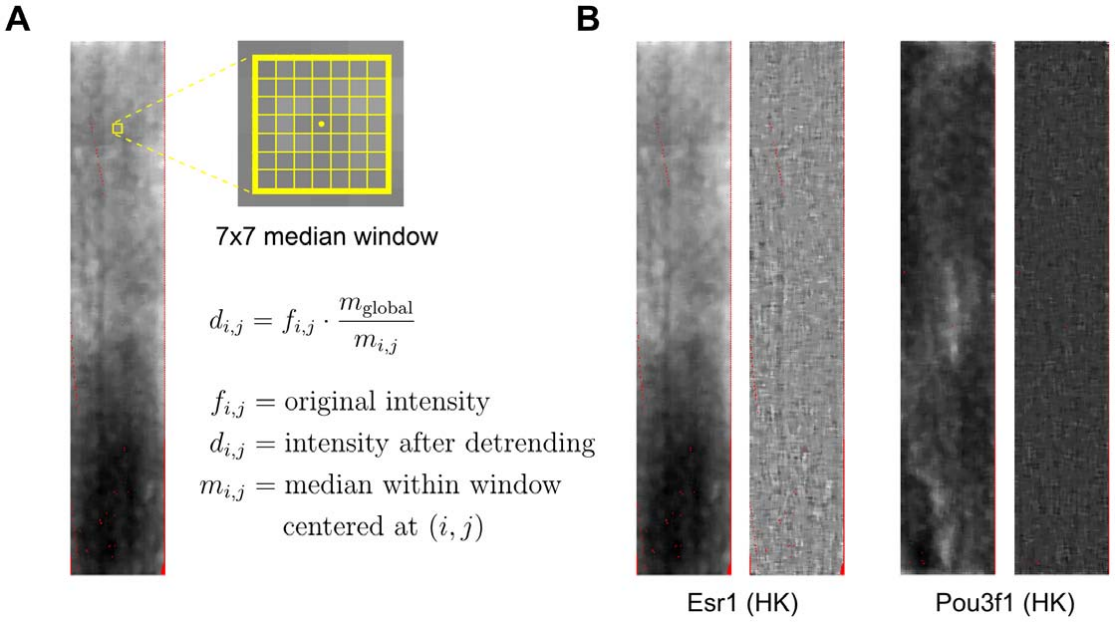
Spatial detrending. (a) A 7×7 median window is used to rescale probe intensities. (b) Two examples of how the spatial detrending step successfully removes large light and dark regions (spatial artifacts) in PBM samples. Original samples on the left, preprocessed samples on the right. Red pixels indicate missing or discarded intensity values.

Next, spatial detrending is performed on the data by rescaling the intensity of each microarray spot by the ratio of the global median and the median calculated within a 7×7 window centered on the spot. This step compensates for the spatial trends (light or dark blotches) often seen in microarray data (Figure 5). A similar spatial detrending step for PBM arrays was previously described by Berger et al. [19].

### Normalization

**Figure 6:**
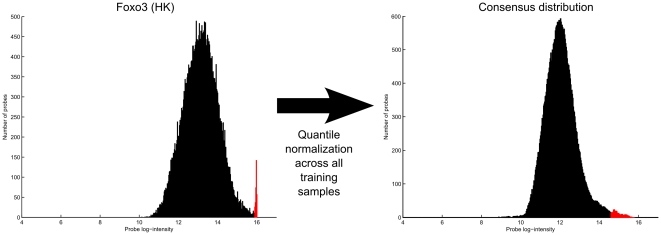
Quantile normalization recovers high intensity tails in saturated samples. The figure shows how the log-intensity histogram of the Foxo3 PBM sample is changed by quantile normalization. An example of how quantile normalization can recover the high intensity tails in saturated PBM samples. The saturated probe intensities (highlighted in red) are recovered by fitting them to the consensus distribution.

In the normalization step, the samples used in learning the motif models are quantile normalized. Quantile normalization assumes that the true intensity distributions (uncontaminated by experimental errors) of different transcription factors have roughly similar shapes. The validity of this assumption is subject to debate, but according to our tests, quantile normalization does improve the accuracy of our model's predictions. We suspect that this improvement is largely due to quantile normalization's ability to recover the high intensity tails in saturated PBM samples (Figure 6). Quantile normalization can also recover samples where an experimental error has resulted in a non-linear monotonic transformation of the probe intensities.

It is critical that we do not simply discard the saturated probes as we did with dark probes, because whereas dark probes can be considered non-informative, high intensity probes are the most informative features in terms of binding affinity. Ideally, the saturated probes would be dealt with by improving the experimental setup and protocol. But in cases where this cannot be done, a computational method is needed.

It is worth noting that the saturation peaks in the intensity histograms are somewhat spread out from the absolute saturation ceiling, so that an ordering exists even for the saturated probes. This ordering may not actually contain any useful information, but if it does, then our quantile normalization step can effectively utilize this information by maintaining the intensity ordering while extrapolating probe intensities beyond the saturation ceiling.

### Probe noise model

In solving our linear system using the ordinary least squares method, we implicitly assume ***ε*** to have a diagonal covariance matrix
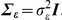
This assumption may not hold, since DNA microarrays have been reported to have roughly linear probe noise 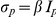 so that the noise level 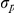 is directly proportional to the probe intensity 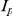 [34]. Hence if technical replicates are available for a PBM experiment, it can be a good idea to estimate the coefficient *_β_* and solve the linear system using a weighted least squares approach
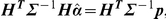
where ***Σ*** is the diagonal noise covariance matrix
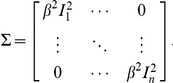



### TF identification

TFs were identified by deducing PWM motifs from the PBM data and comparing the PWMs to mammalian PWMs from TRANSFAC release 2010.2 [35] and JASPAR [36]. First we used the motif discovery tool MEME [21] to build six PWMs with a minimum length of six bases for each TF. We ran MEME using the 500 PBM probe sequences with the highest signal mean for each sample. These signal means were normalized and used as sequence weights for MEME. We observed better results using the weighted sequences than we did without the weights. We let MEME search for motifs using both the forward and reverse complement sequences, and only accepted motifs that occurred in at least 10 training sequences. In addition, we allowed motifs to have multiple repeats within each individual training sequence. After PWMs were deduced, they were compared with existing mammalian binding matrices in TRANSFAC and JASPAR using Tomtom [37] for similarity scoring. We then identified the TFs based on the p-values of the Tomtom results. Additionally, as the databases contained only a few PWMs for each TF family, literature was used to infer exact TF family members.

## Results

### PBM affinity prediction

We assessed the performance of our protein-DNA binding model using PBM data from the DREAM5 transcription factor/DNA motif recognition challenge. The dataset consisted of 86 paired PBM samples for a total of 82 murine transcription factors, each hybridized onto two different PBM platforms (HK and ME). Transcription factors *Mzf1* and *Pou1f1* had two technical replicates in both arrays, while *Zscan10* had three. The two arrays had different probe designs, but both arrays were designed to contain every 10-mer in the sequence of at least one probe on the array. The ME array was designed by Julian Mintseris and Mike Eisen [14], and the HK array by Hilal Kazan, following methodology described by Philippakis et al. [38]. Both PBM platforms were based on Agilent 44K arrays with custom 60-mer probes. Of the total length of each probe, 25 bases were used for the flanking sequences. The PBM array data files in the DREAM5 dataset contained probe intensities for 40 526 probes in the ME array, and for 40 330 probes in the HK array. Both foreground and background intensities were available, but we only used the foreground intensity information.

The goal of the challenge was to predict probe intensities on one array based on intensities measured on the other array. We applied our binding model to the problem by first learning the TF specific affinity vectors 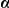 using the training samples (i.e. the PBM samples that were given), and then predicted probe intensities on the target array using that array's design matrix (exact probe sequences for both arrays were known). Since the PBM arrays in the DREAM5 dataset contained only 40K probes, we regularized the underdetermined linear system by only including a subset of all 4–8-mers. We chose to include 2000 7-mers and 1000 8-mers, since we did not see significant improvements in prediction accuracy beyond this number (Figure S2). We also used all 4–6-mers, for a total of 8376 K-mers with lengths between 4 and 8 bases.

**Figure 7:**
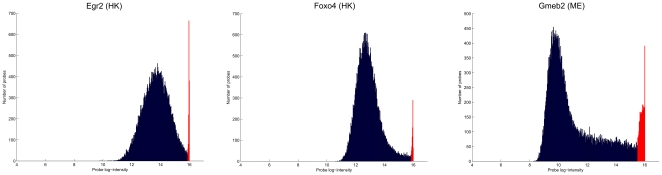
Examples of highly saturated PBM samples. Each figure shows a probe log-intensity histogram where saturated probes are highlighted in red color.

**Figure 8:**
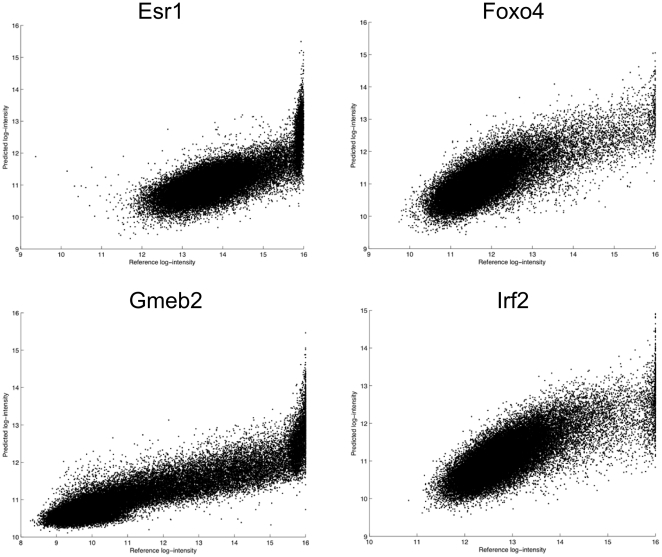
Scatter plots of predicted intensities and saturated reference samples. The y-axis represents predicted probe intensities, while the x-axis represents true probe intensities on the reference array. The scatter plots clearly indicate the negative effect that reference sample saturation has on assessing the accuracy of model predictions.

In some samples, a relatively large number of probes were found to be saturated at high intensities (Figure 7). In total, 22 HK array samples and 13 ME array samples contained such saturation artifacts. We used quantile normalization to deal with saturation in the samples used for training the motif model, but we originally did not perform any normalization on the reference samples against which our predictions were compared. In the DREAM5 challenge, this requirement was enforced by the organizers, who did not grant teams access to the reference samples during the challenge. However, as is clearly evident from the scatter plots of Figure 8, saturation in the reference samples did have a significant effect on reported correlations. Spatial artifacts were also highly abundant in the PBM samples (Figure S1).

**Figure 9:**
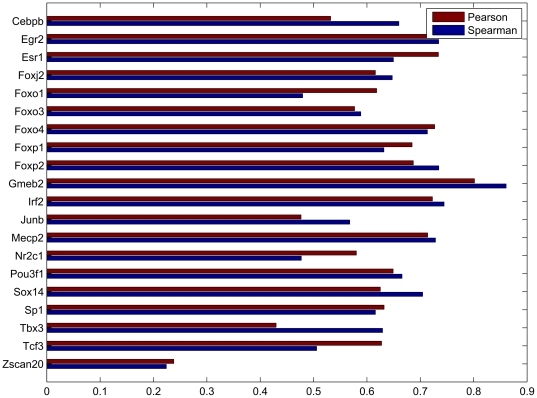
Pearson and Spearman correlations between the predictions of our method (HK→ME) and measured intensities on the ME array. Due to space constraints, results are only shown for the first 20 TFs. Results for all TFs are provided in supplementary Tables S1 and S2.

Using preprocessing and quantile normalization for the training samples only, our model was capable of predicting probe intensities on the target array with average Pearson and Spearman correlations of 0.624 and 0.624, across the 86 paired PBM samples. This placed our method as the best performer in the DREAM5 challenge final ranking. Figure 9 shows the correlation between our model's predictions and measured probe intensities for the 20 first paired PBM samples in the DREAM5 dataset. Full listings of our model's prediction accuracies for all 86 samples are available in supplementary Tables S1 and S2, for HK-to-ME and ME-to-HK predictions, respectively.

After the DREAM5 challenge we also tested the effect of applying preprocessing and quantile normalization to both the training samples and reference samples. The two groups of samples were normalized separately. The result was that the average Pearson and Spearman correlations increased to 0.670 and 0.670, respectively. Although we suspect that these reference-corrected correlations are probably more indicative of the model's true predictive power, we will hereafter only discuss correlations against uncorrected reference samples, analogous to the original DREAM5 performance evaluations.

Berger et al. report a Spearman correlation of 0.53 for their 8-mer E-scores for a single TF across two different PBM array designs. For two technical replicates from a single PBM array they observe a Spearman correlation of 0.91 [19]. The average correlations between our predicted and measured probe intensity correlations are hence higher than those reported by Berger et al. for E-score correlations. We also compared our model against a full 8-mer model where a probe's intensity is predicted based on the highest median intensity K-mer (HMIK) in the probe's sequence. This model was described by Chen et al. in their RankMotif++ paper as a benchmark against which their RankMotif++ algorithm was compared [25]. For all 86 paired samples, our linear model achieved an average Pearson correlation of 0.624 against the HMIK predictor's correlation of 0.515. The average Spearman correlations were 0.624 and 0.418 for our model and the HMIK predictor, respectively.

In their paper, Chen et al. showed that the 8-mer based HMIK predictor performed better than the PWM motif models at predicting binding affinities. The PWMs in these comparisons were derived using a number of PWM discovery algorithms, including MatrixREDUCE, MDScan, PREGO, Seed and Wobble and RankMotif++ [25]. The fact that our model performs better than the HMIK predictor therefore suggests that our model is also more accurate than the PWM based algorithms.

We also assessed the effect of the preprocessing steps on prediction accuracy: averaged correlations across all 86 PBM samples are shown in Table 1. Although the average accuracy only sees moderate improvements from preprocessing, it is worth noting that most samples in the dataset contained only minor artifacts. The samples that did have significant artifacts also saw a stronger boost in accuracy. We also noted that quantile normalization tended to bring the Pearson and Spearman correlations more in line with one another.

**Table 1:** Effect of preprocessing steps on prediction accuracy.

	Original signal	+	Low filtering	+	Spatial detrending	+	Quantile normalization
**Pearson**	0.603		0.603		0.607		0.624
**Spearman**	0.618		0.620		0.623		0.624

***This table shows the effect that different preprocessing steps have on the prediction accuracy of our model. The Pearson and Spearman correlations are averaged over all 86 PBM sample pairs. We used samples from the HK array as our training samples.***

**Figure 10:**
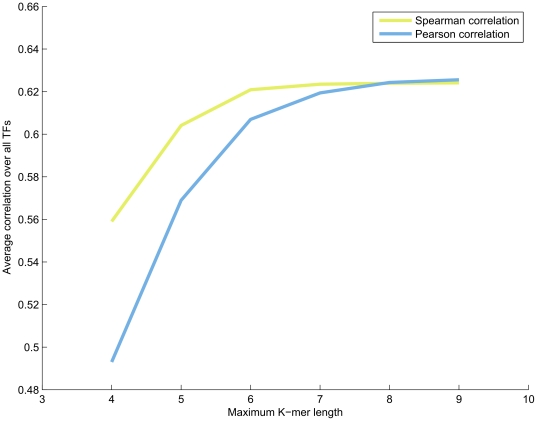
Dependence of model prediction accuracy on maximum K-mer length used in the model. To prevent the linear system from becoming underdetermined, the K-mers in this figure were regularized so that for 7–9-mers, only the 1000 most informative K-mers were included.

The accuracy of our model depends on the maximum length of the K-mers included in the design matrix. Although the additive model allows reasonably good predictions to be made using K-mers as short as 4 bases, the accuracy does consistently improve as the K-mer length approaches 8 bases. No significant improvement is seen from including K-mers longer than 8 bases (Figure 10). We also tried adjusting the number of regularized 7–8-mers included in the model, and found that roughly 1000 highest median intensity 7-mers and 8-mers are enough to achieve saturation in terms of accuracy (Figure S2).

### Examination of affinity scores

**Figure 11:**
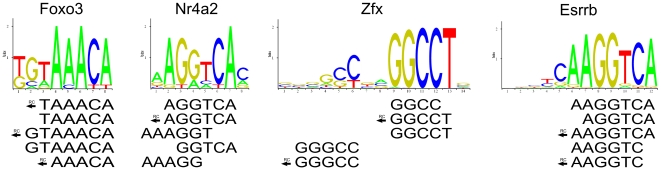
Agreement between top affinity K-mers and JASPAR sequence logos. Shown at the top of the figure are JASPAR Core sequence logos for four TFs. Visible below the sequence logos are the top five highest affinity K-mers from the linear model, for all four TFs. An arrow and the characters “RC” indicate reverse complement K-mers. All sequence logos are for Mus musculus, and were downloaded from JASPAR.

Since our model associates each K-mer with a TF specific binding affinity, we can better understand the binding specificity of a TF by studying its top affinity K-mers. These highest affinity K-mers can then be contrasted with K-mers selected according to median probe intensity. We performed this comparison, and noted that the top median intensity K-mer lists mostly contained 8-mers and hardly any shorter K-mers. We also observed a disproportionally high number of 8-mers containing guanine or cytosine repeats among the top median intensity K-mers. In contrast, among the top affinity K-mers we saw many short K-mers, and less enrichment for the G/C repeats. The top affinity K-mers were also in excellent agreement with the TF binding motifs found in JASPAR Core, even for gapped motifs (Figure 11). The 20 highest linear affinity K-mers for all 86 PBM samples are available in supplementary Table S3. The highest median intensity K-mers can be found in supplementary Table S4.

We further found that the 4-mer affinities learned by the model were significantly correlated across unrelated TFs, with an average Pearson correlation of 0.56. Correlations between technical replicates were even higher, typically in the neighborhood of 0.90. This result implies that even though 4-mer affinities do show variation between TFs, they also have a shared background that may reflect either a systematic artifact in PBM measurements or a common theme in TF binding.

### Probe noise model

We tested the probe noise model by fitting an affine curve to the 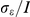 data for the three Zscan10 replicates in the DREAM5 dataset (Figure S3). We then calculated the noise covariance matrix based on a fitted affine relationship, and solved for 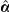 using weighted least squares. Our finding was that the use of the probe noise model had no significant impact on prediction accuracies. This result may be explained by the observed lack of strong correlation between probe noise level and intensity (Figure S3).

### Gapped and reverse complement K-mers

To better handle proteins with a binding specificity for gapped sequences, we experimented with incorporating gapped K-mers into our linear model. We extended our model with all 8-mers with a single nucleotide gap in the middle, and then regularized the gapped K-mers using the same median intensity approach we used for the contiguous K-mers. We found that the inclusion of 500 gapped 8-mers to the model did improve prediction results in a statistically significant manner (p = 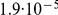, sign test), although the improvements were very small (average Pearson correlation 0.624→0.626). We judged that the added complexity and computation time did not justify the minor improvement in accuracy, although the result did imply that gapped K-mers might be a valid extension to our model.

Next we studied the effect of strand specificity on our model by constraining all reverse complement K-mers to have equal binding affinity contributions. We found that the loss of strand specificity induced by the constraint had a systematic negative effect on prediction accuracies. The average Pearson correlation across all 86 PBM samples dropped significantly from 0.62 to 0.56 (p<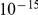, sign test). A full listing of the HK-to-ME prediction accuracies for both strand specific and non-specific models is available in supplementary Table S5. The systematic negative effect may imply a strand specific artifact in PBM arrays.

### TF identification

**Figure 12:**
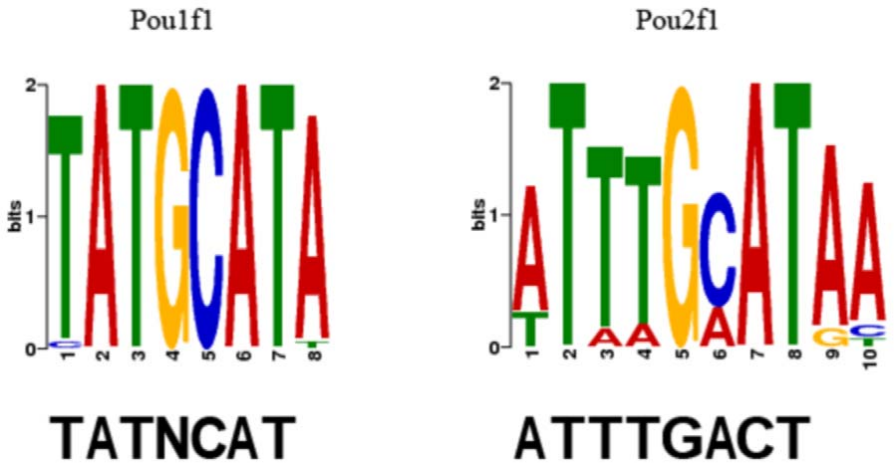
Differentiating between TFs from the same family. At the top of the figure are shown the MEME-predicted sequence logos for Pou1f1 and Pou2f1. Below are shown the binding site consensus sequences from literature [39], [40].

The bonus round of the DREAM5 challenge involved identifying the unnamed transcription factors hybridized to the test PBM arrays. To achieve this, we ran the motif discovery tool MEME and compared the discovered motifs to known mammalian TF motifs in TRANSFAC and JASPAR. However, motif databases contain only a few motifs for each TF family and thus the exact TF name cannot be reliably identified. Thus, if the predicted TF names according to Tomtom were the same for several TFs, we used literature to distinguish the TFs. For example, TFs #13 and #51 in the DREAM5 dataset were both predicted to belong to the POU family of transcription factors. However, *Pou2f1* is known to bind to consensus sequence 5′-ATGCAAAT-3′ [39] while *Pou1f1* favors the consensus sequence 5′-TATNCAT-3′ [40] (see Figure 12). By comparing the conserved motifs to the determined MEME motifs, we were able to correctly identify the TFs. Using our approach we were able to correctly identify seven TFs out of the 66, a result which earned us the first place in the bonus round. Additionally, 15 TFs were identified within the correct TF family. To sum up, even though the computational recognition of TFs is a difficult problem in general, our example demonstrates that it is possible to distinguish TFs within the same family using sequence data.

## Discussion

We have presented a linear model for uncovering TF binding specificity based on PBM measurements. While we only tested our model using data and metrics from the DREAM5 transcription factor/DNA motif recognition challenge, our model can also be applied in less artificial contexts. One obvious application is to use our model for predicting genomic binding sites and their associated TF affinities. This can be achieved by first generating the K-mer affinity vector **α** based on PBM or other data, and then calculating the predicted binding strength at every genomic position by sliding a fixed length window over the genome. Since dsDNA probes on PBM microarrays are not subject to epigenetic effects, the protein's baseline binding affinity toward DNA sequences will be captured. This baseline affinity can then be combined with histone and other epigenetic data to accurately predict cell type specific regulatory interactions. Probabilistic methods and tools for this type of integrative prediction have already been presented in the literature [41]–[43].

Several extensions to our model can be considered. One idea is to enhance our algorithm's normalization step by learning the consensus distribution based on unsaturated samples only, and to then fit only saturated or otherwise aberrated samples to the consensus distribution. A shape-preserving normalization technique would then be applied to the unsaturated samples to bring the rest of the samples to the same intensity scale. Another idea is to learn motif models from ChIP-seq data by placing sequence windows on top of ChIP-seq peaks and using the sequences within those windows to build the design matrix and learn the affinity vector **α**. Due to the *in vivo* nature of the ChIP-seq experiments, this will produce a binding model that attempts to take epigenetic effects into account - at least if those phenomena are somehow correlated with the surrounding DNA sequence.

Another potential extension to our model lies in defining a distance metric for evaluating the similarity of two motifs. Indeed, many metrics have been proposed in the literature for measuring the similarity of PWM motifs: one such example is the Tomtom algorithm by Gupta et al. [37]. Similar metrics could be devised for K-mer based motif models, although the situation is complicated by K-mer regularization, which can cause two motif models to have only a handful of shared K-mers.

One issue with the current model is that the design matrix columns are not independent; for instance, the column for a 4-mer is often a linear combination of four 5-mer columns. This means that the K-mer affinity solutions are not unique. We tried to avoid this issue by first learning a 4-mer model, then learning a 5-mer model based on the residual, then a 6-mer model on the new residual and so forth, but found that our original model (with 10 conjugate gradient method iterations) systematically produced better results (p = 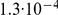, sign test on Pearson correlations). We also tried using only 6-mers and regularized 7- and 8-mers, but again the results were worse than our original model. If unique solutions are desired, one should use the stepwise residual approach, as the decrease in accuracy for that method was relatively mild (average Pearson 0.624→0.614).

One downside of our linear model is the lack of a powerful visual interpretation for the motif model. Due to their mononucleotide-based nature, PWM models can be visualized as graphical sequence logos that are easily interpreted by humans. The same cannot be said of K-mer based models, where the motif can only be described as a set of K-mers toward which a protein has a high binding affinity. The interpretation is made particularly difficult due to the lack of positional information for the motif's constituent K-mers. One way for visualizing K-mer based models would be to convert the model to one or more PWM motif models that would attempt to encode the same specificities as the K-mer model.

One interesting problem in motif modeling is the handling of proteins with gapped motifs, i.e. proteins whose DNA binding motifs contain positions where the nucleotide content does not matter. PWM models can handle such gaps by giving low weights to all mononucleotides inside the gaps. Our proposed K-mer model does not weigh individual nucleotides within K-mers, and hence does not model gapped motifs in this way. Instead, gapped motifs are modeled as a sum of the affinity contributions of the K-mers found before and after the gap. A potential advantage of modeling gapped motifs in this manner is that the length of the gap is not rigidly constrained. This allows the model to accommodate proteins that bind with motifs of variable gap size. On the other hand, this makes our model less powerful at handling proteins for which the gap size is rigidly constrained. It is also important to ensure that the sequences for which the design matrices are built are not too long, so that the K-mer constituents of a gapped motif are constrained to be reasonably close to one another in the sequence.

In conclusion, our linear K-mer based motif model represents a departure from traditional PWM based motif models, and was the best performing method in the DREAM5 transcription factor/DNA motif recognition challenge. Based on our own measurements, the model exhibits significantly higher performance than the full 8-mer model described by Chen et al., while producing more compact motif model representations. This suggests that K-mer based motif models may provide a practical and powerful alternative to mononucleotide models.

## Supporting Information

Figure S1 Examples of PBM samples with spatial artifacts. Red pixels indicate missing intensity values.
(TIF)

Figure S2 Effect of the regularized K-mer count on prediction accuracy.
(TIF)

Figure S3 Probe noise modeling. The figure shows a scatter plot of the relationship between average probe intensities and sample standard deviations, across three Zscan10 PBM replicate samples. Also shown is the least squares linear fit to the data.
(TIF)

Table S1 Prediction accuracies for all 86 sample pairs (HK→ME). Legend: This table contains the full prediction accuracy assessments for all 86 PBM sample pairs and 7 different prediction models. Included among the 7 models are the highest median intensity K-mer (HMIK) predictor, and 6 versions of the linear prediction model with different preprocessing steps.
(XLS)

Table S2 Prediction accuracies for all 86 sample pairs (ME→HK). Legend: This table contains the full prediction accuracy assessments for all 86 PBM sample pairs and 7 different prediction models. Included among the 7 models are the highest median intensity K-mer (HMIK) predictor, and 6 versions of the linear prediction model with different preprocessing steps.
(XLS)

Table S3 Top 20 highest affinity K-mers for all 86 HK array samples. Legend: This table lists the top 20 highest affinity K-mers for each of the 86 HK array samples. The K-mers were learned using the linear model with low intensity probe filtering, spatial detrending and quantile normalization enabled.
(XLS)

Table S4 Top 20 highest median intensity K-mers for all 86 HK array samples. Legend: This table lists the top 20 highest median intensity K-mers for each of the 86 HK array samples. By the median intensity of a K-mer we mean the median intensity across all probes that contained the K-mer. This table is provided for the purposes of comparing with Table S3.
(XLS)

Table S5 Comparison between strand specific and non-specific models. Legend: This table lists the prediction accuracies for both the strand specific and non-specific models, for all 86 paired PBM samples. The predictions were made in the HK→ME direction.
(XLS)
