# Supplementary figures and images for: A Linear Model for Transcription Factor Binding Affinity Prediction in Protein Binding Microarrays
Source: PLoS One. 2011 May 26;6(5):e20059. doi: 10.1371/journal.pone.0020059 (PMC3102690; doi:10.1371/journal.pone.0020059)

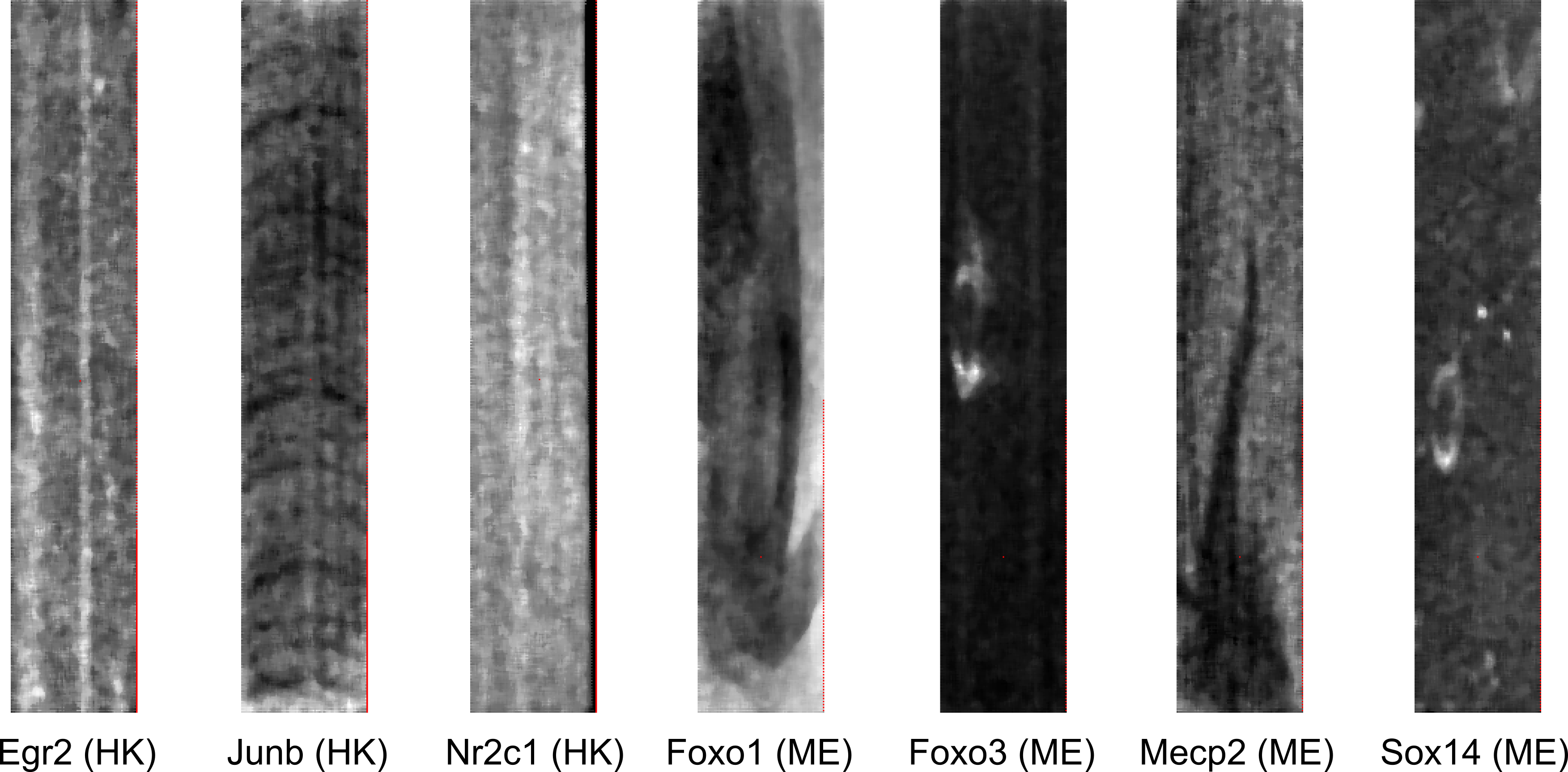

Supplement: Figure S1 — Examples of PBM samples with spatial artifacts. Red pixels indicate missing intensity values. (TIF) [file pone.0020059.s001.tif]

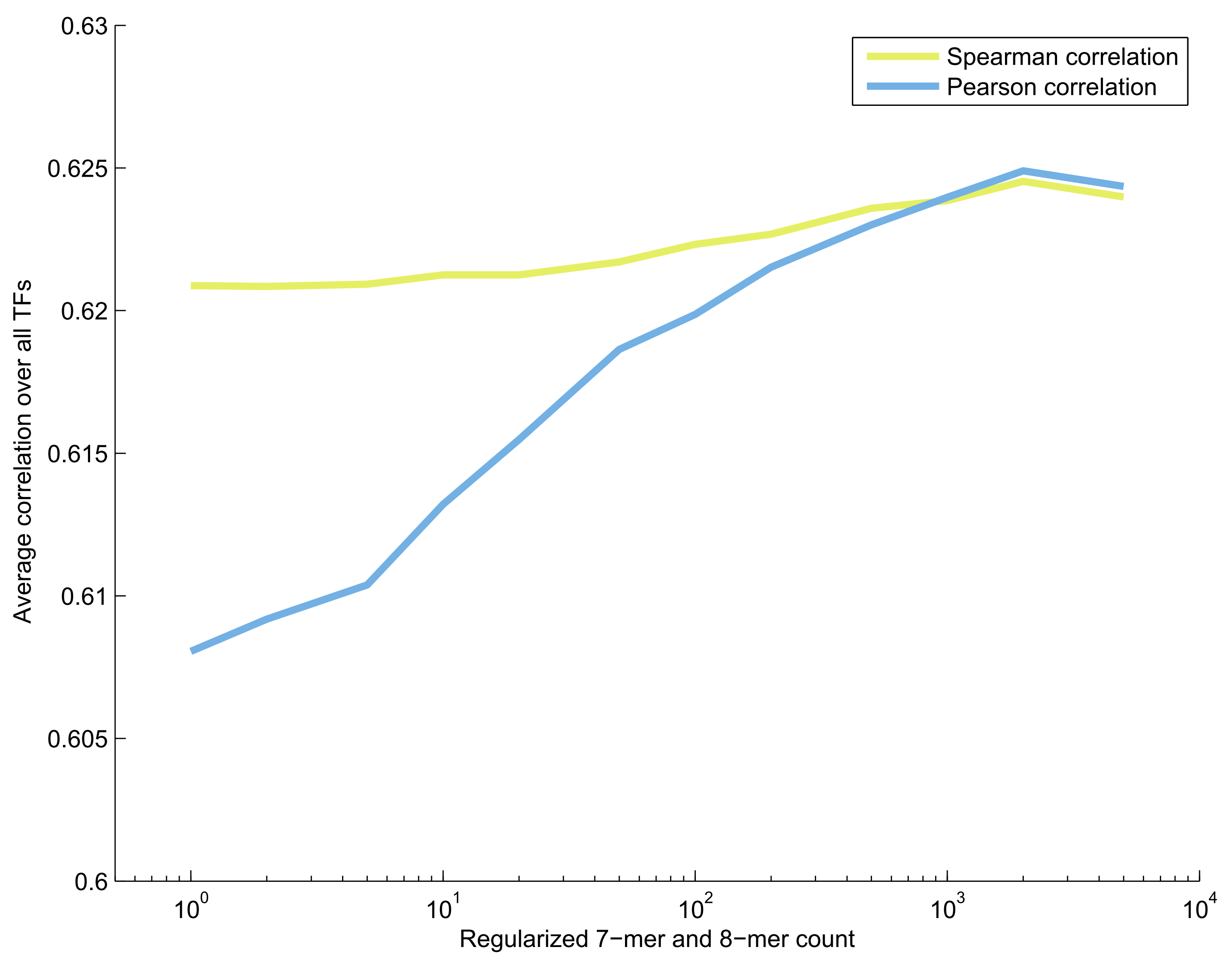

Supplement: Figure S2 — Effect of the regularized K-mer count on prediction accuracy. (TIF) [file pone.0020059.s002.tif]

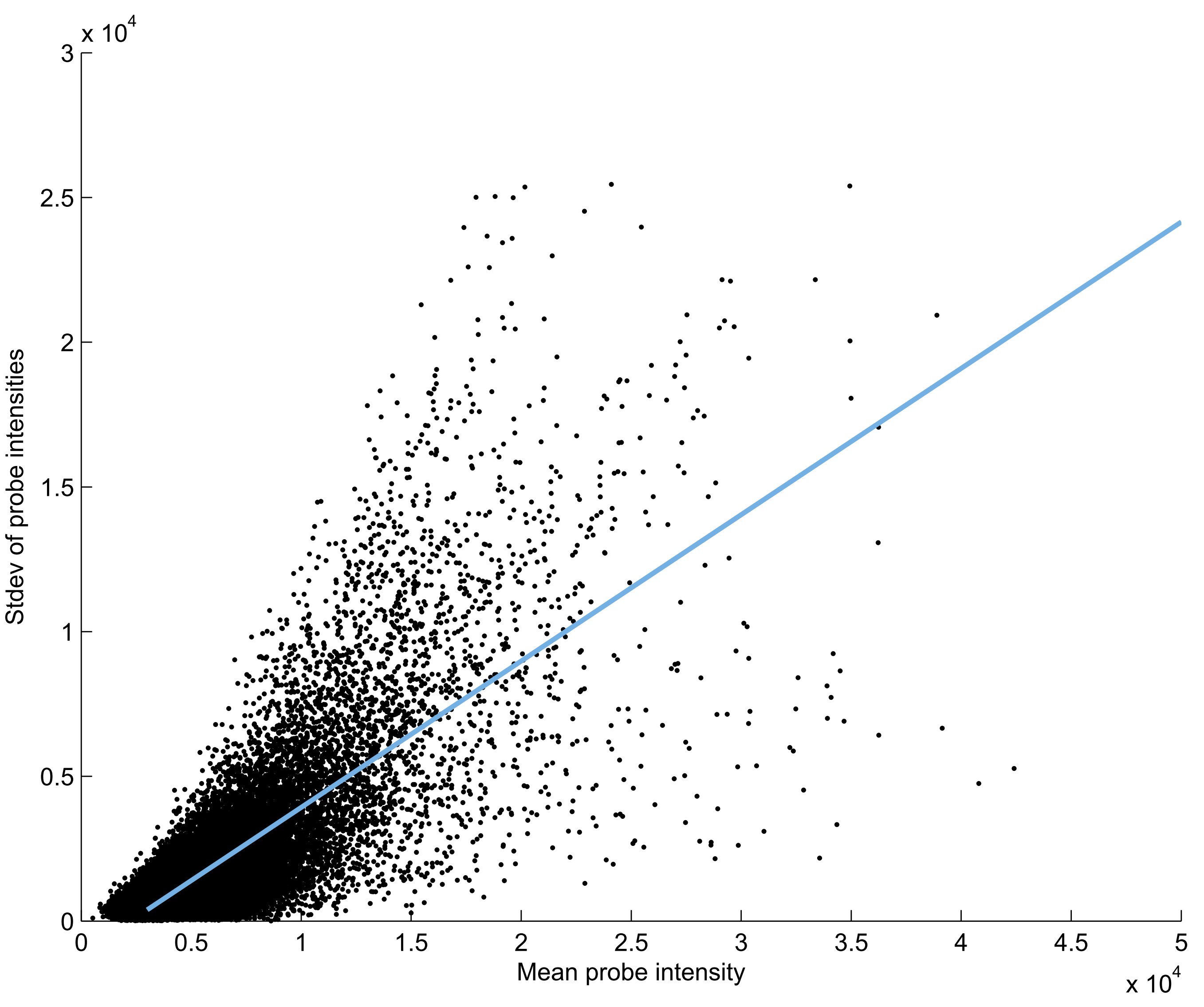

Supplement: Figure S3 — Probe noise modeling. The figure shows a scatter plot of the relationship between average probe intensities and sample standard deviations, across three Zscan10 PBM replicate samples. Also shown is the least squares linear fit to the data. (TIF) [file pone.0020059.s003.tif]
